# Promoting validation and cross-phylogenetic integration in model organism research

**DOI:** 10.1242/dmm.049600

**Published:** 2022-09-20

**Authors:** Keith C. Cheng, Rebecca D. Burdine, Mary E. Dickinson, Stephen C. Ekker, Alex Y. Lin, K. C. Kent Lloyd, Cathleen M. Lutz, Calum A. MacRae, John H. Morrison, David H. O'Connor, John H. Postlethwait, Crystal D. Rogers, Susan Sanchez, Julie H. Simpson, William S. Talbot, Douglas C. Wallace, Jill M. Weimer, Hugo J. Bellen

**Affiliations:** ^1^ Department of Pathology, Penn State College of Medicine, Hershey, PA 17033, USA; ^2^ Institute for Computational and Data Sciences, Pennsylvania State University, Park, PA 16802, USA; ^3^ Department of Molecular Biology, Princeton University, Princeton, NJ 08540, USA; ^4^ Department of Molecular Physiology and Biophysics, Baylor College of Medicine, Houston, TX 77007, USA; ^5^ Department of Molecular and Human Genetics, Baylor College of Medicine, Houston, TX 77007, USA; ^6^ Department of Biochemistry and Molecular Biology, Mayo Clinic, Rochester, MN 55906, USA; ^7^ Mouse Biology Program, School of Medicinel, University of California Davis, Davis, CA 95618, USA; ^8^ Department of Surgery, School of Medicine, University of California Davis, Davis, CA 95618, USA; ^9^ The Jackson Laboratory, Genetic Resource Science, Bar Harbor, ME 04609, USA; ^10^ Department of Medicine, Brigham and Women's Hospital and Harvard Medical School, 360 Longwood Avenue, Boston, MA 02215, USA; ^11^ California National Primate Research Center, University of California Davis, Davis, CA 95616, USA; ^12^ Department of Neurology, University of California Davis, Davis, CA 95616, USA; ^13^ Department of Pathology and Laboratory Medicine, University ofWisconsin-Madison, Madison, WI 53711, USA; ^14^ Institute of Neuroscience, University of Oregon, Eugene, OR 97403, USA; ^15^ School of Veterinary Medicine, University of California Davis, Davis, CA 95616, USA; ^16^ Department of Infectious Diseases, College of Veterinary Medicine, The University of Georgia, Athens, GA 30602, USA; ^17^ Department of Molecular, Cell and Developmental Biology, University of California, Santa Barbara, CA 93117, USA; ^18^ Department of Developmental Biology, Stanford University, Stanford, CA 94305, USA; ^19^ Department of Pediatrics, Children's Hospital of Philadelphia, Philadelphia, PA 19104, USA; ^20^ Pediatrics and Rare Diseases Group, Sanford Research, Sioux Falls, SD 57104, USA; ^21^ Department of Molecular and Human Genetics, Neurological Research Institute (TCH), Baylor College of Medicine, Houston, TX 77007, USA

**Keywords:** Model organisms, Technology, Human diseases, Omics, Integration, Phenomics, Research resources, Validation

## Abstract

Model organism (MO) research provides a basic understanding of biology and disease due to the evolutionary conservation of the molecular and cellular language of life. MOs have been used to identify and understand the function of orthologous genes, proteins, cells and tissues involved in biological processes, to develop and evaluate techniques and methods, and to perform whole-organism-based chemical screens to test drug efficacy and toxicity. However, a growing richness of datasets and the rising power of computation raise an important question: How do we maximize the value of MOs? In-depth discussions in over 50 virtual presentations organized by the National Institutes of Health across more than 10 weeks yielded important suggestions for improving the rigor, validation, reproducibility and translatability of MO research. The effort clarified challenges and opportunities for developing and integrating tools and resources. Maintenance of critical existing infrastructure and the implementation of suggested improvements will play important roles in maintaining productivity and facilitating the validation of animal models of human biology and disease.

## Teaser

**Summary:** Model organisms have the ability to illuminate human biology and disease. Here, we discuss strategies proposed during recent virtual discussions organized by the NIH for improving the rigor, validation, reproducibility and translatability of model organism research.

## Introduction

Model organisms (MOs) have played a central role in modern biology (Nurse, 2021). In addition to providing insights into fundamental biological processes, MOs are able to facilitate clinical diagnosis, provide insight into the molecular and cellular mechanisms of rare and common human diseases (Baldridge et al., 2021; Moulton et al., 2021; Giansanti and Fraschini, 2019), and contribute to drug discovery (Konno et al., 2020). Invertebrates such as the fruit fly *Drosophila melanogaster* and the nematode worm *Caenorhabditis elegans* have orthologs of many human genes (Yamamoto et al., 2014; Shaye and Greenwald, 2011). Although not all genes are conserved, those that are provide elegant tools to study fundamental and broadly shared biological mechanisms in health and disease (Bellen et al., 2010; Markaki and Tavernarakis, 2020). We are reminded, however, by a 100-year-old truism, “each character [phenotype] is affected by many genes and each gene affects many characters [phenotypes]” (Wright, 1934), that phenotypes are central to understanding life and disease.

As many MO researchers study diverse aspects of human biology, the National Institutes of Health (NIH) led by the Office of Research Infrastructure and Programs (ORIP) recently facilitated discussions related to (1) the maintenance and development of tools and resources for MO research (Box 1); (2) strategies for developing ways to predict the impact of therapeutic interventions; (3) approaches to harness phenotypic diversity in MOs; and (4) strategies for mining the massive volume of MO data being generated. Summaries of these ‘Virtual Workshops on Validation of Animal Models and Tools for Biomedical Research’ are available at https://orip.nih.gov/about-orip/workshop-reports. The discussions focused on the central aim of potentiating a better understanding of human biology and disease. Key challenges in MO research identified during these discussions included the maintenance, improvement and addition of stock centers and centers for data integration and phenotyping. Given the various strengths of MOs, a clear consensus emerged that tools and resources should continue to be developed, broadly and in a coordinated manner. The importance of studying diverse MOs became clear during the sessions listed in Table S1. Furthermore, numerous less commonly used MOs with specific experimental strengths have proven useful for addressing a wide range of research questions (Box 2).

**Box 1. Model organism (MO) tools and resources for biomedical research**

Improving strategies to humanize MOs to better model human diseases is an important goal of this field. Humanization of individual genes can be achieved by replacing the MO gene with the human ortholog. Humanization allows the assessment of the functional impact of rare variants that may cause Mendelian diseases (Bellen et al., 2019; Moulton et al., 2021), many of which are shared across MOs (Lenffer et al., 2006).

Reference atlases representing normal and disease anatomy, histology, pathology and cell biology are needed to allow improved comparison between MO and human data (Macy and Horvath, 2017). Furthermore, some of the most promising benefits to disease model research are expected to come from the exploding field of spatial biology, which involves the anatomical anchoring of high-throughput and economical, multiscale, high-content, spatially resolved, molecular analysis, including transcriptomics (Waylen et al., 2020), proteomics and metabolomics.

Antibodies are extremely useful reagents as they can determine where proteins are localized in the cell and organism, and can be used to perform immunoprecipitations to identify interacting proteins. The lack of good antibodies for many proteins in many species has been a recurrent problem as most antibodies generated by companies are designed against human proteins. Nanoantibodies are an alternative that offers numerous advantages. They are single-chain immunoglobulins that are only ∼15 kDa, and their small size and good binding properties allow numerous novel approaches including higher-quality immunoprecipitation (Neumüller et al., 2012). Because they are genetically encodable, nanobodies can be fused with functional domains of other proteins, expressed by a transgene, and used to determine protein localization, to degrade a protein, to re-localize a protein or to trap a protein in the extracellular space *in vivo*, in a cell-specific manner (Caussinus et al., 2012; Vigano et al., 2021). More extensive nanobody libraries should be created for MOs to allow numerous *in vivo* manipulations.

Similarly, proximity labeling methodologies allow imaging at subcellular resolution, labeling of interacting proteins and identification of proteins within spatially defined cellular domains. Advances in proximity labeling can improve our understanding of signal transduction, transient protein interactions and subcellular proteomes in their native environments in various experimental conditions (Bosch et al., 2021).

Split binary expression systems, including split-GAL4, allow one to label specific cell types with higher resolution than conventional methods. Split inteins allow two fragments of a protein to self-splice back into a full-length functional protein when expressed in the specific cell type by using cell-specific enhancers (Pinto et al., 2020). Further development of split protein technologies to enhance their use in many MOs should be a priority (Luan et al., 2020a,b).

Many drugs target G-protein-coupled receptors (GPCRs) that modulate hormone and neuromodulator signaling. Creating an exhaustive set of genetically encoded GPCR biosensors that span the full range of hormones and neuromodulators found in animals and can be targeted to specific cell types would be another very useful resource (Paek et al., 2017).

Another priority should be developing better *in vivo* gene-tagging strategies with fluorescent proteins for microscopy methods or for metal-binding proteins for micro-computed tomography (microCT)-based methods, to determine gene expression patterns and protein localization. In addition, by including tags that allow the use of Cre/loxP or FLP/FRT to induce mutant tissue that are now labeled with a different marker, one can efficiently create conditional knockouts and conduct mosaic phenotypic analyses with precise *in vivo* genotyping. These methods have so far been developed for flies and fish (Li et al., 2019; Nagarkar-Jaiswal et al., 2017), but other MOs would benefit from adopting these approaches.

National and perhaps regional centers are needed to increase the availability of recently developed, difficult to access, cutting-edge, high-resolution microscopy techniques, and to visualize, analyze and integrate those data. These methods include isotropic multiview light-sheet microscopy, expansion microscopy and focused ion beams (Gao et al., 2019). 3D imaging and modeling of cells and their arrangements into tissues can be approached using serial electron microscopy and microCT/histotomography (Cheng et al., 2011; Hildebrand et al., 2017; Ding et al., 2019). Broad and rapid access to histotomography for centimeter-size samples at submicron voxel resolutions (Yakovlev et al., 2021) will be particularly useful for studying samples of millimeter to centimeter scale.

In large projects, detailed data can be difficult to access by other team members or outside groups, and sharing of data is frequently incomplete or missing even after publication. This problem inhibits validation and broader benefit of research projects, including independent data mining for discovery. Data sharing, quality, accessibility and integrity can be improved through the collection of quality metadata and the use of findable, accessible, interoperable and reusable (FAIR) principles (Wilkinson et al., 2016; Dempsey et al., 2022 preprint).

**Box 2. Strategies to improve validation in less commonly used MOs**

**Rabbits** have long been used for biomedical research, especially for muscular and cardiovascular disorders. Creation of a knockout rabbit consortium would (1) generate and supply genetic rabbit models; (2) allow deep phenotyping of genetic lines; (3) provide training on experimental handling, common surgeries and reagent administration; (4) improve annotation of the rabbit genome and enhance the coverage and accuracy of current genome sequences; and (5) facilitate collaborations between academia and industry (Sui et al., 2018).

**Naked mole rats** live about six times longer than their predicted lifespan based on body size. They also display unique characteristics, including (1) hypoxia and hypercapnia tolerance; (2) imperviousness to acid burn and other painful stimuli; (3) hypo-functioning pain signaling associated with osteoarthritis; (4) unusual immune cell populations; (5) resistance to cancer and cardiovascular diseases; (6) resistance to plaques, tangles, and neurodegeneration associated with beta-amyloid and tau in the brain; and (7) resistance to common aging phenotypes. Public resources needed include species-specific antibodies and numerous molecular and genomic tools (Buffenstein et al., 2020).

**Guinea pigs** are particularly useful as models for assessing infectious disease pathogenesis and treatment efficacy. Guinea pig models mimic human disease on multiple levels, from cellular to systems levels, and share infectious disease characteristics with humans. Persistent barriers against further validation of guinea pigs as models of human disease include technical challenges, such as target doses for therapeutics, lack of species-specific reagents and addressable scientific limitations (Padilla-Carlin et al., 2008).

**Hamsters** show great similarities to humans in their lung, liver and pancreatic physiology, making them especially useful for studying diseases of those organs and, in particular, for viral infections like influenza. In COVID-19 studies, severe acute respiratory syndrome coronavirus 2 (SARS-CoV-2) viral replication has been shown to occur in the respiratory and gastrointestinal tracts, and airborne transmission of SARS-CoV-2 between hamsters has been documented, making them a useful model for the study of transmission and vaccine development (Muñoz-Fontela et al., 2020). Mobilization of technologies commonly used in mice, such as fate mapping and conditional mutagenesis, to hamsters will aid in model development and validation (Muñoz-Fontela et al., 2020).

**Pigs** as models for human disease are utilized for their large size, short gestation, multiple births, continuous mating, similarities of structure and function for the cardiovascular and gastrointestinal systems, and availability of multiple well-characterized breeds. Improved annotation of the swine genome would advance pigs as a disease model. More than 40 genetic modifications to swine have been created to overcome immune barriers to xenotransplantation, and more are being produced to facilitate xenotransplantation of human tissues into swine. Pigs are also being used for testing of neuroprotective and regenerative cell therapies that could be applied to traumatic brain injury and other central nervous system models. Current needs include improved standardization and development of magnetic resonance imaging, visualization tools and atlases. Behavioral tests for pig models being developed require further validation (Perleberg et al., 2018).

**Large animals**, such as dogs, pigs and horses, collectively share several hurdles, including limited access to proper husbandry, limited numbers of trained staff and lack of tissue banks. In addition, more-effective use of large-animal models would come from expanding the use of magnetic resonance imaging and computed tomography, promoting standardized methodologies and reporting, developing molecular reagents for species, identifying naturally occurring models, and promoting training for veterinarians and future researchers in the complexities of using large-animal models for research. Current needs include extensive housing facilities, research networks, imaging core facilities, genetic core facilities and tissue repositories for genomics and phenomics, as well as large-animal core facilities (Hotham and Henson, 2020).

**Small aquarium fish**, including zebrafish, medaka, swordtails and platyfish, stickleback, cavefish and mummichog, provide easily accessible and effective models for human disease. The zebrafish, which presents 82% of human disease genes in its genome (Howe et al., 2013), has been used to identify more genes by forward genetic screens than any other vertebrate. Small aquarium fish are excellent models for diseases due to the ease of performing reverse genetics, making transgenic individuals, conducting drug screens, testing susceptibility to infectious diseases, and the ability to make germ-free and defined-microbiome individuals. These fish can effectively model diseases ranging from congenital malformations (Grant et al., 2017) to degenerative diseases like diabetes (Riddle et al., 2018), communicable diseases like tuberculosis (Prouty et al., 2003; Swain et al., 2006), behavioral conditions like autism (Ruzzo et al., 2019) and microbiome-affected diseases. The utility of small aquarium fish as models for human disease would be enhanced by strong continuing support for the Zebrafish Information Network to expand to non-zebrafish piscine models of human disease by standardizing gene nomenclature and ortholog identification. The utility of these MOs can also be expanded by supporting stock centers to house different important fish species, improving efforts to humanize fish models, better technologies for making knock-ins, the establishment of centers to facilitate imaging and genome editing, and the development of nanobodies designed to work on all fish model species. Support of programs that facilitate collaborations between clinicians and fish disease model researchers needs to be encouraged and standardizing phenotyping across species tied to human phenotypes is key to this (Bradford et al., 2017).

**Aquatic models other than fish**, including amphibians and sea slugs, provide special opportunities to investigate mechanisms of human disease. Axolotls and frogs provide exceptional models for tetrapod embryogenesis and, especially, insights into ancestral mechanisms of body part regeneration that the human lineage has lost. Sea slugs provide models for complex behaviors driven by simple nervous systems and accessible neurons. Several investments would improve contributions from these models to human health, including a centralized amphibian genomic and phenotypic database that would identify orthologs tied to other species, unify gene nomenclature, curate gene expression patterns and identify cell types orthologous to those of humans. Additional support for improved tools for knock-ins and other reverse genetic methodologies would spur medical model development. Continued support for stock centers is especially important for aquatic models (Ponomareva et al., 2015).

## Forward and reverse genetic screens

Study of the full range of phenotypes caused by genetic mutations or drugs is a key aspect of disease model research that can be improved to enhance our understanding of human disease mechanisms. Forward genetics involves the generation of, ideally, random mutations, followed by the identification of mutations that affect a specific biological process or phenotype of interest. In contrast, reverse genetics selects a list of genes in which mutations are generated or their expression is decreased, followed by phenotypic analyses to gain insight into gene function. The conservation of genetic pathways in metazoa, combined with the speed and tractability of microscopic phenotyping, made both forward (St Johnston, 2002; Jorgensen and Mango, 2002) and reverse genetic screens in invertebrate MOs such as *D. melanogaster* and *C. elegans* highly informative for probing the function of human disease genes (Bellen et al., 2019; Apfeld and Alper, 2018; Baldridge et al., 2021; Verheyen, 2022). Interestingly, as a result of evolutionary conservation, invertebrate mutant phenotypes in tissues or organs with no obvious human homologs can shed light on human disease mechanisms (Washington et al., 2009).

Reverse genetics involves the generation of collections of mutants in targeted genes using increasingly powerful tools. Today, most approaches take advantage of clustered regularly interspaced short palindromic repeats (CRISPR) technology (Khalil, 2020) for genome editing, which can and should be expanded beyond their initial use to simply create loss-of-function mutants, or ‘knockouts’. CRISPR can also be used to generate mutations that are allelic orthologs to specific human disease mutations, to functionally inhibit gene/protein function in particular tissues for assessment of cell- and tissue-specific phenotypes, and to assess expression patterns and subcellular protein localization. CRISPR technology has been used to create multiple disease models by insertion of the yeast *GAL4* gene in flies (Lee et al., 2018; Kanca et al., 2019, 2021), worms (Wang et al., 2017a) and, to some extent, mice (Clark et al., 2020). Embryonic lethal mutant phenotypes may be pursued using cell- or tissue-specific knockouts, which are more easily applied to invertebrates and zebrafish, the externally growing embryos of which allow the facile study of mutant phenotypes at cell resolution prior to death.

The external development of zebrafish embryos makes them particularly well suited to another form of reverse genetics, genetic knockdowns with antisense morpholinos (Nasevicius and Ekker, 2000; Stainier et al., 2017), with which gradations of phenotype can inform gene function in a way that is equivalent to allelic series of mutants (Nasevicius and Ekker, 2000). Their power and limitations have been well characterized by the zebrafish community (Stainier et al., 2017). Furthermore, the gene specificity of knockdowns in antisense morpholinos is validated using different target sequences for the same gene, for instance, by altering splice junctions versus blocking translational initiation (Lamason et al., 2005). They have also been used in combination with allele-specific mRNA rescue to study targeted phenotypes (Tsetskhladze et al., 2012). In sum, genetic knockouts and knockdowns in zebrafish and other organisms can be used to provide complementary reverse genetic insight into the mechanisms of human biology and disease.

## Systematic phenotyping across MOs

We know that a comprehensive understanding of any gene's function or a chemical's effects on an organism is only possible with in-depth phenotyping, including behavioral, physiological, cellular, molecular or tissue effects. Two vertebrate systems showcase complementary approaches. The zebrafish system was established through primarily phenotype-first screens (Amsterdam and Hopkins, 2006; Mullins et al., 2021), where in-depth biological analyses of genetic mutations predated molecular cloning. The Knockout Mouse Phenotyping Program (KOMP2) project provides a complementary, gene-first model for phenotyping practices (Brommage and Ohlsson, 2019). The KOMP2 is the US component of the International Mouse Phenotyping Consortium (IMPC) that aims to knockout and functionally characterize all protein-coding genes in the mouse genome (Cacheiro et al., 2019). All IMPC sites adhere to a uniform phenotyping pipeline that includes protocols, data collection and reporting standards. Data are available via a data portal (www.mousephenotype.org), and knockout mice are available from public repositories (e.g. www.mmrrc.org). A combination of one or both approaches, where genotype/phenotype is clearly connected in a central database using shared ontogeny, represents a robust future approach for disease research in MOs.

Indeed, the systematic collection of comprehensive datasets and making them accessible will be essential to allow comparisons between different MOs and human data to probe mechanisms of gene action relevant to human disease. Phenotypic and genotypic data for each MO phenome project should ideally include the sequence of the genome, single-cell sequencing data of whole organisms or specific tissues, a metabolomic and proteomic profile, and, in particular, complete computational measurements, rather than just qualitative description, of cellular and tissue phenotypes based on imaging technologies (Box 1).

The breadth of phenotypic inquiry should also include behavior, fertility, diurnal rhythmicity tests, mating behavior, sleep, sensitivity to heat and mechanical shock, phototaxis and the full extent of physiological responses (Neckameyer and Bhatt, 2016). For anatomical phenotypes evident from single time points, permanent collections of embedded mutant animals and tissues with corresponding genetic and phenotypic data in central repositories could greatly facilitate community-based validation and discovery (Cheng et al., 2016). These approaches will require extensive coordination between laboratories of different expertise, the involvement of clinicians to ensure clinical relevance, and the sharing and availability of standardized reagents and protocols.

Variation in disease phenotypes with age and developmental stages in humans needs to be considered when designing studies in MOs. Chemical and retroviral mutagenesis-based forward genetic screens in zebrafish and mice have led to the identification of numerous mutations that affect vertebrate embryonic development (Amsterdam et al., 2004; Nüsslein-Volhard, 2012; Fuentes et al., 2018; Guénet, 2004; Kile and Hilton, 2005). Many genetic and toxicological approaches in zebrafish focus on embryonic phenotypes, owing to the transparency, small size and experimental accessibility of embryos. While embryos have yielded numerous insights into gene function, many human diseases do not cause symptoms until juvenile or adult stages. This means that the next phase of disease modeling will need to include genetic and chemical screens capable of detecting behavioral, morphological, cellular, molecular, and other phenotypes in juveniles and adults (Pelegri and Mullins, 2016; Gray et al., 2021). For example, forward genetic screens in adult mice isolated mutants that are highly relevant to understanding sleep disorders in humans (Funato et al., 2016).

It is worth emphasizing that disease modeling using forward and reverse genetic approaches in MOs takes into consideration that orthologous cell types and organs affected in human disease may not be easy to identify or even present in the MO. Individual phenotypes, or more often, sets of phenotypes caused by mutations in orthologous genes in different organisms with different phenotypes have been called phenologs (McGary et al., 2010). For example, the *patched* gene that affects wing vein pattern in flies via the Hedgehog signaling pathway causes Gorlin syndrome and basal cell carcinoma in humans (Gailani et al., 1992). Despite great differences in anatomy and microanatomy between species, underlying molecular and cellular mechanisms of disease are often conserved. To validate the connection between pathways and phenotype, it will be important for MO tools to include facile ways to compare mutant phenotypes between MOs and humans. For example, MO phenologs extending to yeast (McGary et al., 2010; Lehner, 2013) have been used to screen drugs affecting molecular pathways involved in human disease (Cha et al., 2012).

The effects of environment on phenotype are important to study in MOs for multiple reasons. Environmental factors such as lifestyle are well recognized to play a major role in the most common causes of disease in modern society (Ezzati et al., 2004; https://www.cdc.gov/nceh/tracking/topics/LifestyleRiskFactors.htm). In addition to the clinical relevance, experimental results and the reproducibility of results when using MOs can be affected by nutrition, viruses, microbiome and other aspects of environment (for topics see Table S1).

The ecological community of commensal, symbiotic and pathogenic microorganisms likely play a causative role in many cases of reproducibility failures, especially in inbred strains of mice. Ancillary husbandry practices such as feces banking, for example, may help in understanding issues associated with gut microbiota. Options for optimization include maintenance of wild mice in containment facilities, use of wild mouse microbiota and controlled exposure to targeted pathogens. Related factors that should be included in publications are strain sourcing, degree of inbreeding of mouse strains and housing conditions. Genetic drift has been noted to affect the gene pool and disease frequency over time in inbred strains and should therefore be monitored as well (Mooser et al., 2018).

In summary, the rise of systematic phenotyping, including a conversion of tissue phenotyping from qualitative to quantitative science, a vast expansion of molecular phenotyping, and integration of these phenotypes across normal and diseased MOs and humans, will be both important and more possible with the development of new technologies. Systematic annotation, integration, and facile access to molecular and cellular phenotypes are needed so that bioinformatic approaches and artificial intelligence (AI) can be used to better understand gene function and disease mechanisms in MOs.

## Mining the genetics of natural variation

Just as humans develop a wide range of genetic and genetically impacted diseases, numerous vertebrate species offer a wealth of opportunities to study the relationship between genes and disease mechanisms (Table S1; Bradford et al., 2017). Mining natural genetic variation in adult MOs, including companion animals and non-human primates (NHPs), can be very valuable for studying disease phenotypes relevant to human diseases.

Evolutionary mutant models (EMMs) are species that allow the study of phenotypes associated with human disease by focusing on mutations that have undergone adaptive selective pressure. Studying how these variants in EMMs cause phenotypes that are pathogenic in humans can suggest avenues for novel disease therapies (Beck et al., 2021). Fish EMMs are particularly abundant and useful because the clade is ancient, the populations are large, and they have global distribution across habitats of vastly disparate temperature, salinity, light and water pressure. For example, natural selection for widely different pigment morphologies for mate recognition in hybrid *Xiphophorous* fish species led to the discovery of naturally occurring melanomas associated with new combinations of pigment gene controls. Studying these fish led to identification of the RTK–RAS–RAF–MAPK pathway (Lemmon and Schlessinger, 2010), critical in the development of human melanoma a decade before it was detected in humans (Wellbrock et al., 2005). The highly variable food opportunities for Mexican cavefish result in binge feeding and starvation, leading to metabolic adaptations that have provided insights into human insulin-resistant diabetes (Riddle et al., 2018). Furthermore, EMMs with superior antiviral defenses, such as pangolins, bats and naked mole rats, are useful for studying transmissible diseases like COVID-19 [the disease caused by severe acute respiratory syndrome coronavirus 2 (SARS-CoV-2) infection] (Stegmann, 2021). In summary, EMMs related to different organ systems provide a fertile ground for probing human diseases (Beck et al., 2021).

Orthologous variants to those associated with human disease are commonly observed in non-human primate colonies. The extremely high conservation between humans and non-human primate genes means that studying these disease variants in these models will better inform us of the function of these genes in human disease. This approach can be pursued by whole-genome sequencing of animals kept and bred in primate centers (Warren et al., 2020; Abbott et al., 2019). Breeding some of these animals may allow phenotypic analyses of variants and drug testing based on the principles of repurposing U.S. Food and Drug Administration (FDA)-approved drugs for use in humans (Boguski et al., 2009), saving time and effort needed to generate gene modification-based disease models.

Mice are often used to model human diseases, but, unlike most patients, they are typically inbred and lack genetic diversity. Valuable insight can be gained by studying single human disease-causing mutations in different genetic backgrounds in mice. For example, genetic variation between mouse strains was found to profoundly modify the impact of human Alzheimer’s disease (AD) mutations on the genetic, transcriptomic and phenotypic recapitulation of human AD (Neuner et al., 2019). Other MOs in which human-like genetically driven disease models spontaneously appear include companion animals, such as cats and dogs taken to veterinary clinics (Gurda et al., 2017).

In sum, the wide range of MOs, whether associated with adaptation to unusual environmental niches or by stochastic appearance as a result of the genetic variation in large-animal populations, provides a rich opportunity to identify series of genes that contribute to the molecular and genetic pathways affected in human disease.

## Cross-phylogenetic, or ‘vertical integration’

**Fig. 1.**
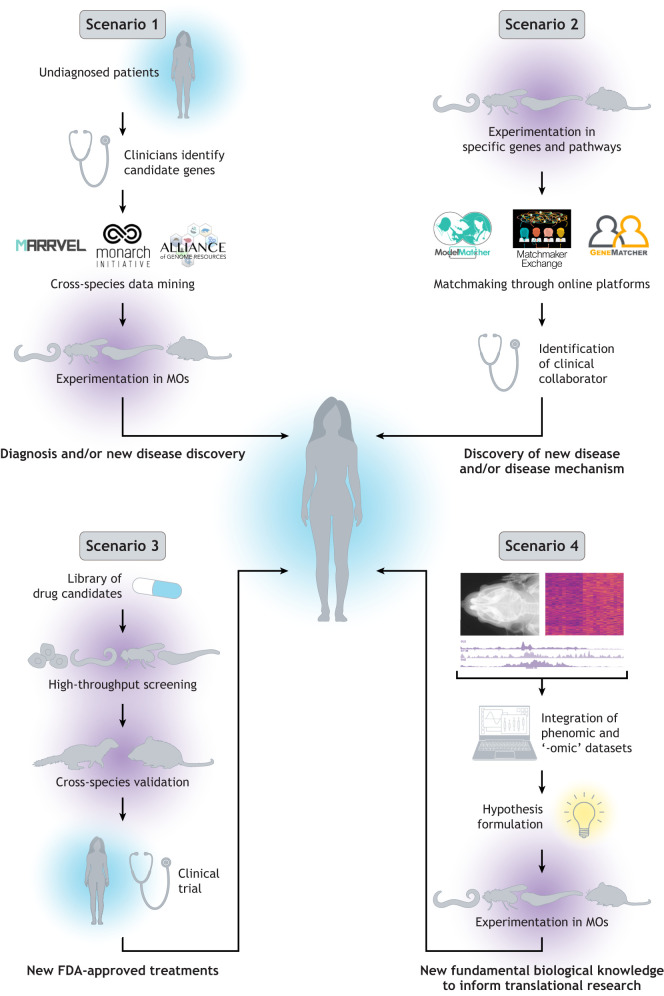
**‘Vertical integration’ facilitates collaborative research between scientists and clinicians with shared interests.** Four different scenarios of vertical integration discussed in the main text are depicted here. The logos of MARRVEL, Monarch Initiative, Alliance of Genome Resources, ModelMatcher, Matchmaker Exchange and GeneMatcher were obtained from respective websites. FDA, U.S. Food and Drug Administration; MO, model organism.

Validation of disease models was a targeted goal of the NIH meetings. How can we improve the value of MOs for diagnostic purposes and elucidate the biological and disease mechanisms to aid in drug discovery? One valuable way to address these goals is through the integration of studies among different species, which we call ‘vertical integration’ (Fig. 1). Cross-phylogenetic data access is needed to allow comparisons between MO and human data to probe and validate mechanisms of gene action relevant to human disease. This contrasts to ‘horizontal integration’ of data from different disciplines such as genetics, biochemistry and cell biology within a single MO or mutant. Both forms of integration require collaborative effort between researchers with common interests and different areas of expertise, as well as the sharing and availability of standardized reagents and protocols. The involvement of translational scientists and physicians is needed to enhance the clinical relevance of vertical integration.

An especially fruitful example of vertical integration is the collaborative efforts among clinicians and MO scientists to participate in the diagnosis of rare disease patients (Fig. 1, Scenario 1). Variants in genes that may cause disease in patients are explored by mining existing data collated in databases like MARRVEL (Wang et al., 2017b) and Monarch Initiative Explorer (Shefchek et al., 2020). MARRVEL is gene centric and collates data from numerous human genetic databases as well as the most studied genetic MOs in an easily searchable format. The Monarch Initiative is an integrative data and analytic platform that connects phenotypes to genotypes across species. The Alliance of Genome Resources (AGR) is an NIH initiative [National Human Genome Research Institute (NHGRI)] to bring all the information for all MOs into one database that is easy to mine (Alliance of Genome Resource Consortium, 2022). Once potential causative variants have been identified, they can be modeled in MOs to aid in diagnosis (Baldridge et al., 2021). In this scenario, collaborations between basic biologists and physician scientists have proven to be effective in diagnosing rare diseases (Splinter et al., 2018).

A second example of vertical integration stems from MO researchers who are interested in discovering the molecular mechanisms of a developmental or physiological process of human diseases associated with a gene or pathway of interest (Fig. 1, Scenario 2). Through GeneMatcher (Sobreira et al., 2015), Matchmaker Exchange (Philippakis et al., 2015) and ModelMatcher (Harnish et al., 2022), clinicians and MO biologists can explore collaboration opportunities with other researchers who have a common interest in tackling shared questions/problems.

Another form of vertical integration is the use of MOs in drug development (Fig. 1, Scenario 3) and toxicology. FDA-approved as well as unannotated small molecules are most easily and more comprehensively screened in MOs like worms (Hunt, 2017) and flies (Millet-Boureima et al., 2021). One can, for example, start by screening drugs in an invertebrate or immortalized cell line, followed by screening top candidates in a well-characterized, small vertebrate like zebrafish (Cassar et al., 2020) prior to the use of mammals. These can then be followed by studies in mouse models or human patient-derived cells. A significant amount of money can be saved in drug development by using cross-species strategies. Furthermore, tests using MOs can be designed to be more humane and less expensive, and to necessitate the use of fewer mammals.

Environmental toxicology work envisioned within the restricted domain of cell culture studies (Thomas et al., 2018) is now re-entering the realm of animal-based toxicology that currently focuses on molecular analyses, using worms, flies, *Daphnia*, zebrafish and frogs, under European leadership (https://cordis.europa.eu/project/id/965406). Ongoing discussions are aimed at finding ways to incorporate the whole-animal morphological phenotyping discussed above. Strong synergies can be expected from the integration of molecular and phenotypic data from drug and environmental toxicology with analogous data from forward and reverse genetic screens using the same MOs.

To take into account the three-dimensional (3D) structure of life, and to make histopathological characterization quantitative, rather than qualitative, histology-like pan-cellular imaging and submicron resolution will be necessary for whole small organisms, and for representative portions of organs of larger organisms. Massive file sizes (tens to hundreds of GB to tens of TB) are required for 3D visualization and analytics at submicron resolutions. It is exciting that the sharing and manipulation of such files is beginning to become possible with improvements in cloud computing. Work towards the systematic annotation, integration, and user-driven correlation of molecular data with multimodal and multiscale cellular and tissue (histopathological) phenotypes would facilitate an unprecedented level of data integration and understanding of human biology and disease.

An interesting strategy for vertical integration is to perform systematic comparisons of gene ortholog knockout phenotypes among different species to provide testable hypotheses and pathogenic mechanisms (Fig. 1, Scenario 4) (Shefchek et al., 2020; Wang et al., 2017a). The cellular composition of all living things inspired a proposal during the ORIP discussions to anchor single cell transcriptomic, proteomic, and metabolomic data (collectively referred to as '-omics') on digital representations of multiscale, pan-cellular, whole organism tissue anatomy and microanatomy. Digital quantification of similar histopathological phenotypes across MOs was also proposed as a unifying and integrative approach to facilitate the modeling of human disease.

One of the most important integrative efforts in the biological sciences is the collective community goal of comprehensively characterizing all functional non-coding genome sequences. These Encyclopedia of DNA Elements (ENCODE) projects focused largely on human, *C. elegans*, *D. melanogaster* and mouse (ENCODE Project Consortium, 2012; Celniker et al., 2009; ENCODE Project Consortium et al., 2020). DANIO-CODE is an inspiring bottom-up community effort of more than 30 laboratories that has integrated published and unpublished zebrafish epigenomic data (Baranasic et al., 2022; Lawson, 2022). An integrated analysis of more than 1800 remapped data sets from 15 distinct developmental stages using chromatin immunoprecipitation with sequencing (ChIP-seq; Robertson et al., 2007), assay for transposase-accessible chromatin with sequencing (ATAC-seq; Buenrostro et al., 2013), RNA sequencing (Emrich et al., 2006), cap analysis gene expression (CAGE; Shiraki et al., 2003), high-throughput chromosome conformation capture (Hi-C; Lieberman-Aiden et al., 2009) and circularized chromosome conformation capture sequencing (4C-seq; Zhao et al., 2006) yielded over 140,000 predicted regulatory elements associated with development and organogenesis. Previously unknown control elements without sequence similarity were discovered in fish and mouse genomes using new tools based on phylogenetic synteny. This work sets the stage for an exciting new possibility – to gain functional insight into the vertebrate epigenome by the systematic CRISPR-Cas-based targeting of vertebrate epigenetic elements using zebrafish, the size and experimental pliability of which facilitate whole-organism phenotyping.

Finally, integration was recognized in the ORIP technology discussions to be a ‘big data’ problem that will require the use of AI as well as accessible scalable cloud-based storage and computing. The challenge is huge because of the different MOs and disciplines involved, each of which uses different terminology. Ontological work will be key to the successful use of AI, as will efforts to render currently subjective and descriptive phenotypic analyses more quantitative and computationally accessible. Integration and facilitation of validation and discovery will require sophisticated data storage, coordination, computation, machine learning and analytics that will require the development of national and regional centers of expertise.

The current professional realities of clinical care make it important to specifically promote collaborative efforts between physicians and physician scientists, who study specific diseases, and MO scientists, who use different species and experimental approaches. The clinical perspective is crucial for guiding bioinformatic strategies to add translational value to work in MOs by drawing phenotypic parallels between human and MOs.

## Conclusions

### Forward and reverse genetics

Forward and reverse genetic studies focused on mutants comprise the heart of the value of MOs and are becoming increasingly sophisticated and enriched in terms of gene and protein targeting, expression studies and other molecular analytics. Their extensions, married to the new technologies and integrations noted below, can be expected to greatly improve their potential impact.

### Resources and new technologies

An essential component of improving the use of MOs is to maintain and significantly expand critical resources and means of data analytics to facilitate the study of basic biology and disease mechanisms. The return on investment of new technological approaches based on new forms of imaging and molecular analytics, and the integration of the derived data is expected to be huge (Box 1).

### Systematic phenotyping

Ideally, collections of mutants can be phenotyped systematically, including minimal essential datasets for cross-species comparison. Comprehensive phenotyping would include high-content molecular technologies correlated with cellular and tissue phenotype. Molecular analytics include transcriptomics, single-cell sequencing, proteomics and metabolomics. Optimally, molecular phenotypes are considered in the context of whole-organism anatomic and cellular morphology. With the arrival of new imaging technologies, tissue phenotyping, currently reliant upon qualitative analysis, now must become increasingly computational, reproducible and quantitative. Broad access to the resulting data will allow the systematic comparison of different cell types and tissues across organisms. The creation of comprehensive, mathematically defined maps of 3D anatomic and cellular morphology of MOs and humans would facilitate systematic comparisons of phenotypes in health and disease. Regional or national imaging centers will be needed for complex imaging technologies, analytics and integrations that require extensive equipment, storage, computation or expert support. New computational approaches will be needed to systematically generate, organize and mine the phenotypic data across species to gain integrative insights from forward and reverse genetic and other approaches.

### Natural variation

Natural variation in MOs can provide unique insight and new models of human disease. Many examples in fish, mice and other species, such as dogs, cats, mole rats and primates, argue that numerous genetic modifiers of disease loci remain to be defined. Genetic studies in these and other species can be used to better define this genetic complexity and identify variants in genes or their regulatory regions that either suppress or enhance disease-associated phenotypes. These studies will be important as they can mimic complexities featured in outbred human genomes.

### Integration

The seminars and discussions organized by the NIH established that improving integration across disciplines and MOs should be a priority. Numerous MOs have unique strengths, and we should therefore maintain this breadth as each MO also has inherent weaknesses. However, collaborations between MO researchers and physician scientists should be encouraged. Furthermore, informatic-assisted optimization of integration between MO phenotypes and the full range of text- and image-based clinical data would help to ensure the relevance of experiments to human health. This would be greatly facilitated by the development of computational approaches to mine existing and new data across MOs and between genetic and toxicological phenotypes.

### MOs and the COVID-19 pandemic

During the COVID-19 pandemic, an extraordinary level of interdisciplinary, multi-organismal, international and multi-agency collaboration among MO researchers yielded key elements of our understanding of SARS-CoV-2 pathogenesis, as well as components needed to accelerate vaccine development (Muñoz-Fontella et al., 2020). These achievements illustrate what can be accomplished through MO research, new technology development, AI and integration of research disciplines.

### MO research and society

The increasingly existential nature of today's global challenges, including the COVID-19 pandemic, climate change, and toxic waste, all point to the unprecedented relevance and urgency of developing the capabilities of disease model research. Successful integration of the richness of MO data will benefit from incorporation of human data as we take systems approaches to understand the genetic and environmental determinants of biology and disease. Perhaps the most important level of data integration that needs to be addressed is to add international and interagency interoperability between data generated in the largely separate realms of disease modeling, toxicology, disease diagnostics, regulatory agencies and, urgently today, climate change. Research reproducibility (Rodgers and Collings, 2021) and impact will be maximized by the proposed maintenance and enhancements in MO tools and resources, data integration and mechanisms of validation.

## Supplementary Material

10.1242/dmm.049600_sup1 Supplementary information
