# Supplementary material for: Promoting validation and cross-phylogenetic integration in model organism research
Source: Dis Model Mech. 2022 Sep 20;15(9):dmm049600. doi: 10.1242/dmm.049600 (PMC9531892; doi:10.1242/dmm.049600)
Supplement: Supplementary information [file dmm-15-049600-s1.pdf]

Table S1. Topics related to modeling and developing drugs for specific diseases/pathologies

Topic	Presenter	Link
Zebrafish Models of Genetic Epilepsies: Challenges and Opportunities	Gerald Downes	https://orip.nih.gov/sites/default/files/Validation_Session_II_Meeting_Report_Final_508. pdf
Zebrafish Models Validate Undiagnosed Human Diseases	Monte Westerfield	https://orip.nih.gov/sites/default/files/Validation_Session_II_Meeting_Report_Final_508.pdf
Forward and Reverse Genetic Approaches in Zebrafish to Understanding Scoliosis	Lilianna Solnica-Krezel	https://orip.nih.gov/sites/default/files/Validation_Session_II_Meeting_Report_Final_508.pdf
Zebrafish as Models for Infectious Diseases	Lalita Ramakrishnan	https://orip.nih.gov/sites/default/files/Validation_Session_II_Meeting_Report_Final_508.pdf
A New Era for Rare Disorders	Rebecca Burdine	https://orip.nih.gov/sites/default/files/Validation_Session_II_Meeting_Report_Final_508.pdf
Humanized Mouse Models for Cardiovascular Regenerative Therapeutics	Kenneth Chien	https://orip.nih.gov/sites/default/files/Validation_Session_III_Meeting_Report_Final_508. pdf
The Pig Stroke Model: Evaluating Neuroprotective and Regenerative Therapies	Franklin West	https://orip.nih.gov/sites/default/files/Validation_Session_IV_Meeting_Report_Final_508.pdf
Therapeutic Development in the Canine Models for Duchenne Muscular Dystrophy	Peter Nghiem	https://orip.nih.gov/sites/default/files/Validation_Session_IV_Meeting_Report_Final_508.pdf
Companion Animal Models of Chronic Pain	Duncan Lascelles	https://orip.nih.gov/sites/default/files/Validation_Session_IV_Meeting_Report_Final_508.pdf
A Rabbit Model of Duchenne Muscular Dystrophy for Preclinical Therapeutic Testing	Renzhi Han	https://orip.nih.gov/sites/default/files/Validation_Session_V_Meeting_Report_Final_508.pdf
Use of Guinea Pig Models to Assess the Efficacy of Enteric Countermeasures	Hailey Weerts	https://orip.nih.gov/sites/default/files/Validation_Session_V_Meeting_Report_Final_508.pdf
Genetic Modeling in the Ferret to Study Disease Pathophysiology, Stem Cell Biology, and Genetic Therapies	John Engelhardt	https://orip.nih.gov/sites/default/files/Validation_Session_V_Meeting_Report_Final_508.pdf
A SARS-CoV-2 Hamster Infection Model to Study the Effect of Vaccine Candidates	Johan Neyts	https://orip.nih.gov/sites/default/files/Validation_Session_V_Meeting_Report_Final_508.pdf

Disease Models & Mechanisms • Supplementary information

and Antivirals		
Cavefish as a Model for Natural Resilience to Metabolic Disease	Nicolas Rohner	https://orip.nih.gov/sites/default/files/Validation_Session_VII_Meeting_Report_Final_508.pdf
Data Integration and Validation of Candidate Variants Regulating Sleep and Rhythms in Cells and Mice	John Hogenesch	https://orip.nih.gov/sites/default/files/Validation_Session_IX_Meeting_Report_Final_508.pdf
Translational Tools and Cross-Species Data Integration Approaches for Multi-Drug Regimen Development for Infectious Diseases	Rada Savic	https://orip.nih.gov/sites/default/files/Validation_Session_IX_Meeting_Report_Final_508.pdf

Disease Models & Mechanisms • Supplementary information
